# Dissociation and PTSD in women with a history of childhood sexual abuse: a pilot examination of a specialized inpatient unit

**DOI:** 10.3389/fpsyt.2026.1855046

**Published:** 2026-07-08

**Authors:** Lotem Zvi, Jonathan E. Handelzalts, Danny Horesh, Inbal Shlomi

**Affiliations:** 1 School of Behavioral Sciences, The Academic College of Tel Aviv-Yaffo, Tel Aviv, Israel; 2 Department of Psychiatry, University of Michigan, Ann Arbor, MI, United States; 3 Department of Psychology, Bar-Ilan University, Ramat-Gan, Israel; 4 Department of Psychiatry, New York University Grossman School of Medicine, New York, NY, United States; 5 Merhavim Medical Center, Beer Yaakov, Israel; 6 The Faculty of Medicine, Tel Aviv University, Tel Aviv, Israel

**Keywords:** complex trauma, CSA, dissociation, inpatient, PTSD - posttraumatic stress disorder

## Abstract

**Background:**

Worldwide, there are relatively few specialized inpatient units dedicated to women with histories of childhood sexual abuse (CSA) and comorbid psychiatric disorders. This pilot study examined dissociation and PTSD among women admitted to such a specialized integrative inpatient unit in Israel. We conduct an in-depth analysis of the role of dissociation in these women’s clinical picture, as well as in their treatment response.

**Methods:**

The study included two phases. Phase 1 used a cross-sectional design to assess the complex inter-relationships between PTSD and various facets of dissociation in women with CSA histories admitted to a specialized inpatient unit (*N* = 108). Phase 2 focused on a sub-sample of participants (N = 28) who completed the inpatient program and completed admission and discharge assessments. Measures included the PTSD Checklist for DSM-5 (PCL-5) and the Dissociative Experiences Scale (DES-II).

**Results:**

In Phase 1, dissociative symptoms were positively correlated with overall PTSD severity and all PTSD symptom clusters, with the dissociative sub-measure of Absorption showing the strongest associations. In Phase 2, PTSD symptoms significantly decreased following treatment. In line with phase 1, reductions in Absorption were associated with improvements in overall PTSD severity and specifically in Hyperarousal symptoms.

**Conclusions:**

Findings from this pilot study indicate the therapeutic potential of a specialized integrated inpatient unit for women with CSA histories. Importantly, our results indicate that dissociation should be regarded as a major therapeutic target, most notably patients’ tendency for maladaptive absorption. These preliminary results should be expanded upon in larger, controlled clinical trials, further elucidating the role of dissociation and other mechanisms of change in similar units.

## 1 Introduction

Childhood sexual abuse (CSA) represents an intensely traumatic experience, often leaving survivors with profound and long-lasting psychological and physical consequences (1). Early exposure to sexual abuse forces the individual to cope with adaptive challenges that far exceed their capacity. The effort to sustain a sense of safety and agency while facing deep helplessness often leads to comprehensive dysregulation within the survivor’s biopsychosocial functioning (2). Clinical frameworks have evolved to capture this breadth of impairment through the construct of complex trauma. Unlike single-event traumas, complex trauma refers to chronic, repetitive experiences that are primarily interpersonal in nature and are characterized by inherent relational power imbalances (3, 4). Such exposure frequently results in a symptomatic presentation that extends beyond the core clusters of posttraumatic stress disorder (PTSD; re-experiencing, avoidance, hyperarousal, negative alterations in cognition and mood (5);). This is formally captured by the construct of Complex PTSD (C-PTSD), which is recognized as a distinct diagnostic category in the 11th edition of the International Classification of Diseases (ICD-11 (6);). This framework recognizes a complex diagnostic profile in which standard PTSD symptoms (re-experiencing, avoidance and hyperarousal) are intertwined with broader systemic dysregulation and unique disturbances in self-organization (6). The present study evaluated the clinical outcomes of an integrative inpatient treatment program for women with histories of CSA, focusing on changes in PTSD symptoms and their associations with dissociative processes. While the majority of participants were diagnosed with standard PTSD alongside various comorbid disorders, the clinical conceptualization of the patients and the unit’s therapeutic approach are deeply rooted in the frameworks of complex trauma. This orientation reflects the recognition that CSA-related trauma is characterized by pervasive developmental, relational, and affective disruptions that necessitate multidimensional treatment (7). Adopting this framework enables the examination and treatment of a shared traumatic core across diagnostically diverse presentations.

Within this framework, dissociation is recognized as a prominent and integral trauma-related response. This central role is formally reflected in the DSM-5 ‘dissociative subtype’ of PTSD, which explicitly addresses persistent symptoms of depersonalization and derealization (5). The clinical significance of these dissociative processes is especially evident in the context of CSA; empirical findings indicate that approximately 30% of women with a history of CSA report severe daily dissociative symptoms (8), a frequency significantly higher than among individuals exposed to other types of traumas (9, 10).

Dissociation is a broad spectrum phenomena, ranging from mild normative experiences to severe clinical manifestations characterized by a lack of integration between mental processes such as consciousness, memory, and perception (5). While such experiences can be a common response to distress, they often evolve into an elaborated and habitual defense mechanism in the face of overwhelming trauma (11). Specifically, in cases of childhood abuse, the severity and frequency of these dissociative responses tend to escalate and manifest through specific pathological dimensions, including amnesia, absorption, depersonalization, or derealization (12). Ultimately, the dissociative processes are often inherently embedded within the core features of post-traumatic symptoms; for instance, the ‘here and now’ quality of re-experiencing and the profound emotional numbing are fundamentally dissociative in nature (13). In a broader clinical sense, these re-experiencing symptoms, alongside hyperarousal, are the clusters most frequently linked to dissociative experiences (14), a connection that can be understood through the lens of emotional modulation (15). Within this framework, hyperarousal and re-experiencing symptoms reflect a state of emotional undermodulation- an overwhelming ‘flood’ of sensory intensity, whereas different dissociation symptoms involve emotional overmodulation, characterized by a defensive ‘shutting down’ of affective states (15, 16). These processes can be viewed as complementary responses to unbearable distress; rather than being a state of simple inactivity, the dissociative ‘hypo-arousal’ is often preceded by, and contingent upon, an acute surge of hyperarousal and perceived threat (17, 18). While this dynamic reflects a general interaction in PTSD, it is especially pertinent for those who have experienced CSA. In these cases, the clinical profile often manifests as a recurring fluctuation between intense activation and defensive disconnection, as dissociation functions as a regulatory strategy to cope with extreme arousal through the hyper inhibition of limbic regions (18).

Despite the documented link between dissociation and PTSD, there is an ongoing debate regarding how dissociation influences treatment outcomes. According to the Emotional Processing Theory (EPT), dissociation might act as a barrier to recovery by inhibiting the emotional engagement necessary for processing traumatic memories (19). Some empirical research supports this concern, linking higher baseline dissociation to diminished symptom reduction (20). However, emerging evidence increasingly challenges the notion of dissociation as a barrier. A recent meta-analysis of 21 clinical trials demonstrated that pre-treatment dissociation was not significantly related to the overall effectiveness of psychotherapy for PTSD, suggesting that standard trauma-focused treatments remain effective even in the presence of dissociative symptoms (21). This is particularly evident among CSA survivors, where significant symptom reduction is consistently observed regardless of baseline dissociative levels (22, 23). Beyond not constituting a barrier, dissociative symptoms appear to be dynamic processes that evolve in concert with other trauma-related symptoms. Both outpatient and specialized inpatient programs have demonstrated that dissociation significantly decreases during treatment alongside reductions in overall PTSD severity (24–26). This indicates interconnected trajectories of change, where improvements in dissociative states and post-traumatic symptoms facilitate one another within the therapeutic process. This recognition aligns with a broader shift toward integrative, pluralistic treatment models for individuals with histories of interpersonal trauma, whose clinical presentations are increasingly conceptualized through the lens of complex trauma (27, 28). These frameworks move beyond single-theory models by combining diverse modalities, ranging from Skills Training in Affective and Interpersonal Regulation (29) and Dialectical Behavioral Therapy (DBT) for PTSD (30) to specialized adaptations of trauma-focused therapies such as Eye Movement Desensitization and Reprocessing (EMDR (31);) and Prolonged Exposure (PE (32);), to meet the heterogeneous needs of this population. Empirical studies conducted in specialized inpatient settings for adults with complex trauma histories following childhood abuse consistently document significant reductions in PTSD symptoms, achieved through differing therapeutic emphases across programs. These include stabilization and the management of self-destructive behaviors within psychodynamic informed trauma treatment (26), group-based interventions focused on emotional processing and interpersonal functioning (33), multimodal approaches integrating psychoeducation with experiential modalities such as movement and art therapy (24), and targeted trauma-processing protocols such as adapted EMDR intervention (25). The current study aims to add to the existing body of evidence by examining both the clinical characteristics and treatment response among women with histories of CSA, treated within a specialized inpatient program. This intensive setting integrates several of the adapted techniques reviewed above, such as DBT for PTSD and somatic grounding, under one roof. Grounded in a relational-psychodynamic foundation, the program combines these stabilization components and tailors them dynamically to each patient’s individual clinical needs, rather than relying on a single uniform protocol. Although the previously reviewed research on inpatient and naturalistic clinical settings consistently focuses on overall symptomatic improvement, considerably less attention has been devoted to examining the interplay between PTSD and dissociation within these settings. In addition, dissociation is a highly complex phenomenon, comprising several aspects and elements (e.g., amnesia, depersonalization, absorption). The examination of these sub-components has been missing from much of the literature, a gap we wish to bridge in our study. Finally, studies that more directly investigate the association between complex trauma and dissociation frequently rely on controlled designs or non-clinical samples that systematically exclude individuals with acute and complex clinical presentations, such as those requiring hospitalization (12, 22). As noted by Hyland et al. (2024) (12), such recruitment strategies tend to underrepresent a population that is particularly critical for examining these associations, and thus hinder external validity. By focusing on women presenting with severe symptomatology and high levels of comorbidity, this preliminary pilot study provides an opportunity to: (a) shed light on the complex interplay between symptoms of dissociation and PTSD among women with CSA. and (b) explore the potential impact of a long-term, integrative inpatient treatment under real-world clinical conditions. The study was conducted in two phases: a cross-sectional phase examining baseline symptomatology and associations between PTSD and various dissociation dimensions, followed by a pilot clinical phase assessing treatment outcome among a sub-sample of women. Beyond describing overall symptom change, the clinical phase of this study tentatively explored the potential role of dissociation within this integrative therapeutic process. At both phases, dissociation was examined both at the global level and across specific dimensions, including amnesia, absorption, and depersonalization/derealization, to explore their differential associations with PTSD symptom clusters among this population.

### 1.1 Research hypotheses

#### 1.1.1 Phase 1

1. Higher baseline dissociation will correlate with greater PTSD severity at admission.

2. Distinct dissociative components will show differential associations with PTSD symptom clusters at baseline.

#### 1.1.2 Phase 2

3. Dissociative symptoms and overall PTSD symptoms will decrease from admission to discharge, including across PTSD symptom clusters, regardless of baseline dissociation levels.

4. Changes in dissociative dimensions will be associated with changes in PTSD symptoms over time, providing a preliminary examination of dissociation-related mechanisms of change. In line with phase 1, here too we expect to find differential associations between change in specific sub-dimensions of dissociations and PTSD.

## 2 Methods

### 2.1 Participants and procedure

The study was conducted at the “By Your Side” psychiatric inpatient department at the Merhavim Mental Health Center, a specialized unit for women with trauma-related psychopathology. While the present study specifically focuses on a subgroup of patients with a history of CSA, the general admission criteria for the unit include being 18 years and older, presenting with at least one of the following: a history of sexual abuse, an eating disorder, or other trauma-related symptomatology. All clinical diagnoses were established by senior psychiatrists following diagnostic assessments and prolonged clinical observation, based on ICD-10 criteria. Hospitalization in the unit is voluntary. Exclusion criteria include severe psychotic states and active substance or alcohol dependence. The clinical model in the department is primarily informed by Herman’s (1992) (4) three-stage model, which organizes treatment around the sequential goals of safety and stabilization, trauma processing, and reconnection. Conceptually, the approach is further grounded in the Theory of Structural Dissociation of the Personality (34), aiming to promote integration among dissociated self-states. Treatment is delivered by a specialized multidisciplinary team comprising psychiatrists, clinical psychologists, social workers, arts therapists, occupational therapists, dietitians and nursing staff. This care is provided through a combination of individual and group-based interventions, with an emphasis on a tailor-made treatment approach, allowing for clinical flexibility in addressing the particular challenges and pace of each patient’s process. To accommodate the diverse fabric of the Israeli female population, the unit provides a culturally sensitive environment that routinely adapts its clinical care to women from various cultural and religious sectors, including secular, orthodox, and Arab backgrounds, among others. The overall length of the program is flexible and clinically driven, determined by individual therapeutic progress rather than a fixed number of sessions. In the initial stabilization phase, symptom management is supported through DBT-PTSD (30). This approach integrates dialectical principles with elements of compassion and mindfulness to specifically target emotion regulation deficits and maladaptive coping strategies, such as self-harm and disordered eating behaviors. By balancing behavioral change with metacognitive awareness and self-compassion, the model aligns with Fisher’s (2017) (35) neurobiologically-informed approach on transforming the patient’s internal relationship with themselves by replacing shame with a compassionate connection to even the most “dis-owned” self-states. As the treatment progresses, these structured modalities are embedded within a relational-dynamic psychotherapy framework (36, 37), in which the therapeutic relationship serves as a central mechanism for addressing attachment disruptions and facilitating psychological integration. This dynamic core is integrated with Sensorimotor Psychotherapy (38) and other somatic and functional interventions, including movement-based and occupational therapies. These “bottom-up” methods address the embodied nature of trauma by working directly with bodily sensations to resolve trauma-related somatic patterns and support daily functioning. Together, this coordinated and flexible treatment model addresses both acute symptom severity and underlying disruptions in self-organization. Regarding the pharmacological context of care, patients routinely entered the unit with established community-based medication regimens. Throughout the course of hospitalization, these regimens were dynamically tailored and optimized based on evolving clinical presentations, adhering to a clinical rationale of maintaining the minimum effective dosage and number of concurrent medications. Pharmacotherapy predominantly featured selective serotonin reuptake inhibitors (SSRIs) targeting post-traumatic symptomatology. Additionally, targeted protocols for anxiety and sleep disturbances were frequently implemented, alongside the selective use of mood stabilizers to address affective dysregulation.

Between 2019 and 2025, women admitted to the unit who reported a history of childhood sexual abuse (CSA) during their initial intake assessment were invited to participate in the study. CSA was defined as abusive or exploitative sexual contact or activity occurring prior to the age of 18, involving a caretaker, an authority figure, or an individual at least five years older than the victim, in situations where a power imbalance precludes the possibility of legal or psychological consent. Participation was voluntary, anonymous, and independent of the treatment process. Ethical approval for the study was obtained from the Merhavim Mental Health Center Institutional. The study was conducted in two phases:

#### 2.1.1 Phase 1: cross-sectional associations between study variables

During the first two weeks of admission, research assistants contacted the participants to coordinate the completion of self-report questionnaires via the Qualtrics platform (39). This phase established the baseline clinical and demographic profile of the full study sample, comprising 108 women with a history of CSA. Of these, 14 participants did not complete the self-report symptom questionnaires. Because symptom data were entirely unavailable for these individuals, missing-data imputation was not considered appropriate. Consequently, the final sample for the Phase 1 symptom analyses consisted of 94 participants. Baseline comparisons indicated no significant demographic or clinical differences between participants who completed the questionnaires and those who did not.

Participants ranged in age from 18 to 60 years (*M* = 29.47, *SD* = 9.55), and all provided initial demographic and clinical characteristics (see **Table 1**). Based on a diagnostic threshold of 33 on the PCL-5, all participants met the criteria for a probable PTSD diagnosis. For participants who were readmitted during the study period (*n* = 6), only data from the most recent hospitalization were included.

**Table 1:** Sample demographic and clinical characteristics.

	n	%	M	SD
Demographics				
Age (years)	108	—	29.47	9.55
Education (years)	108	—	13.38	2.44
Monthly Income (ILS)				
0-4,000	57	58.2	—	—
4,000-8,000	18	18.4	—	—
8,000-12,000	121	12.2	—	—
> 1,2000	11	11.2	—	—
Marital Status				
Single	69	63.6	—	—
Married	20	18.7	—	—
Divorced	11	10.3	—	—
Prior Psychiatric Hospitalizations	108	—	4.83	5.12

#### 2.1.2 Phase 2: a pilot examination of treatment effectiveness

The second phase was designed as a clinical pilot study exploring the effectiveness of treatment at the specialized inpatient unit. This phase included a smaller sub-sample of the phase 1 sample (28 participants). Independent t-tests were conducted to compare the baseline profiles of participants who were included in Phase 2 against those who were not. The analyses revealed no significant differences between the two groups regarding baseline dissociation, PTSD severity, or demographic variables.

Since this is a first-ever examination of the unit’s clinical model, it was important to include only those who met several criteria, to achieve a complete and valid evaluation. This, we included women who: (a) expressed sufficient interest and motivation in participating in a pre-post treatment evaluation. (b) were hospitalized at the unit for at least one month without interruption or intermittent discharges. (c) adhered to the basic treatment setting (attending most treatment sessions and group activities, undergoing continuous psychiatric follow-ups). These thresholds were intended to ensure sufficient exposure to the unit’s integrative therapeutic framework. The mean duration of hospitalization for this group was 4.5 months (*SD* = 2.48, range = 1–12 months). To assess clinical change, participants in this pilot group were asked to complete the same battery of self-report measures at the point of discharge that they had completed upon admission. Although paired dissociation data were available for 28 participants, PTSD data were complete for 25; therefore, correlational analyses were conducted on the 25 participants with complete data across both measures. This pilot sample was characterized by high clinical complexity: 85.7% of participants presented with more than one clinical diagnosis, and 46.4% were diagnosed with three or more concurrent conditions (e.g., eating disorders, borderline personality disorders, mood disorders). Furthermore, participants reported an average of 3.3 lifetime suicide attempts. Demographically, the pilot subsample had a mean age of 28.80 years (*SD* = 9.22) and an average of 13.10 years of education (*SD* = 2.20). The majority of participants in this phase were unmarried (60.7%) and reported a low monthly income level of 0–4,000 ILS (57.1%).

### 2.2 Measures

All measures used in this study were validated Hebrew versions of the original scales (40, 41).

#### 2.2.1 Demographics

Participants were asked to provide demographic and background information, which included age, years of education, income level, marital status and hospitalization history (both general and specifically at the “By Your Side” facility) (See **Table 1**).

#### 2.2.2 Post-traumatic stress symptoms

The PTSD Checklist for DSM-5 (PCL-5) (42) is a 20-item self-report measure assessing PTSD symptoms experienced over the past month. Items are rated on a 5-point Likert scale ranging from 0 (not at all) to 4 (extremely), yielding a total score between 0 and 80, with higher scores indicating greater PTSD severity. A cut-off score of 31–33 is commonly used to indicate probable PTSD (43). The items are organized into four symptom clusters corresponding to DSM-5 criteria: Re-experiencing (Cluster B), Avoidance (Cluster C), Negative alterations in cognition and mood (NACM) (Cluster D), and Hyperarousal (Cluster E). Internal consistency was ω = .87 at admission and ω = .93 at discharge.

#### 2.2.3 Dissociative symptoms

The Dissociative Experiences Scale (DES-II) (44) is a 28-item self-report screening measure assessing dissociative symptoms. Participants rate the frequency of each experience on a scale ranging from 0% to 100%. Beyond the total score, the scale assesses three distinct factors that capture different dimensions of the dissociative experience: Amnesia, reflecting gaps in memory and awareness; Depersonalization/Derealization, involving a detached sense of self or reality; and Absorption, representing an altered state of focused attention and internal preoccupation. The total score is calculated as the mean of all items, with scores of 30 or higher indicating clinically significant dissociative symptomatology (45). Internal consistency was *ω* = .93 at both admission and discharge. At the subscale level, internal consistency at admission and discharge, respectively, was as follows: amnesia (ω = .81, ω = .89), absorption (ω = .80, ω = .80), and depersonalization/derealization (ω = .83, ω = .80).

### 2.3 Statistical analysis

Data were analyzed using IBM SPSS Statistics (version 28). Descriptive statistics were used to summarize demographic and clinical characteristics of the sample. In the first phase of the study, Pearson correlations examined associations between dissociative symptoms, total DES-II and subscales, and PTSD severity at admission, including the PCL-5 total score and symptom clusters. To evaluate changes over the course of treatment in the second phase, paired-samples *t* tests were conducted to assess within-subject differences between admission and discharge. Two- sided *p* values were reported and effect sizes were calculated using Cohen’s d based on the standard deviation of the difference scores. A mixed-design ANOVA was performed to examine whether PTSD symptom reduction was influenced by initial dissociation levels. Participants were categorized into high and low dissociation groups via a median split of baseline DES-II scores, and the time-by-group interaction was tested to determine if improvement trajectories differed by initial dissociation severity. To examine synchrony of change, change scores (Δ) were calculated for all measures by subtracting discharge scores from admission scores. Pearson correlations were then used to assess associations between reductions in the total and subscale dissociative scores (ΔDES-II) and reductions in PTSD severity (ΔPCL-5).

To control for multiple comparisons across the four PTSD symptom clusters, a Bonferroni correction was applied, resulting in a significance threshold of α = .0125. Similarly, a Bonferroni correction was applied for the three DES-II subscales, resulting in an adjusted threshold of α = .0167.

## 3 Results

### 3.1 Phase 1: baseline interrelationships between dissociation and PTSD symptoms

In this part of the study, we set out to examine the complex inter-relationships between PTSD and dissociation. To this end, we examined the associations between both the total scores and the specific subscales of our two primary measures: the PCL-5 and the DES-II, which include the dissociative factors of Absorption, Depersonalization/Derealization, and Amnesia. **Table 2** presents the Pearson correlations between these main study variables.

**Table 2:** Pearson correlations between PTSD symptoms and dissociation at admission.

	DES-II Total	Absorption	Depersonalization\Derealization	Amnesia
PCL-5 Total	.520***	.552***	.450***	.336***
Re-experiencing	.393***	.444***	.314**	.285**
Avoidance	.28	.36	.24	.07
NACM	.449***	.584***	.477***	.364***
Hyperarousal	.370***	.513***	.530***	.355***

*n* = 94. NACM , Negative Alterations in Cognition and Mood. **p <.01. *** *p* <.001.

As can be seen in **Table 2**, a significant positive correlation was found between total dissociative symptoms and overall PTSD severity, indicating that higher levels of dissociation were associated with more acute post-traumatic distress. A more detailed examination of the dissociative factors revealed a consistent pattern of associations with most PTSD symptom clusters, though the strength of these relationships varied across dimensions.

The Absorption dimension emerged as having the most robust and consistent associations with PTSD symptomatology, with the strongest relationship in the entire correlation matrix observed between Absorption and the NACM cluster. High levels of Absorption were also strongly linked to the Intrusion and Hyperarousal clusters. To further examine this pattern, Steiger’s *Z* tests were conducted to compare the correlation coefficients of Absorption across the different PTSD clusters. Results revealed that the correlation between Absorption and the NACM cluster was significantly stronger than its correlation with both the Intrusion (*p* = .049) and Avoidance (*p* = .019) clusters, though it did not significantly differ from its correlation with the Hyperarousal cluster (*p* = .20).

The Depersonalization/Derealization factor also showed significant associations with these same PTSD clusters, though these correlations were more moderate in strength. In contrast, the Amnesia factor exhibited the weakest associations among the dissociative dimensions, showing a lower magnitude of correlation across the various PTSD domains. To statistically compare the relative strength of these associations with overall PTSD severity, Steiger’s *Z* tests for dependent correlation coefficients were conducted. Results indicated that the correlation involving Absorption was significantly stronger than the correlation involving Amnesia (*p* = .001), and marginally stronger than the correlation involving Depersonalization/Derealization (*p* = .052). While significant correlations were widespread across the Intrusion, NACM, and Hyperarousal clusters, such associations were not observed with the Avoidance cluster. Across all significant results, the associations were consistently more prominent with the NACM and Intrusion clusters. Finally, analyses indicated that baseline symptom severity was not significantly correlated with demographic variables, including age, education, or marital status.

### 3.2 Phase 2: pilot clinical study

**Figure 1:**
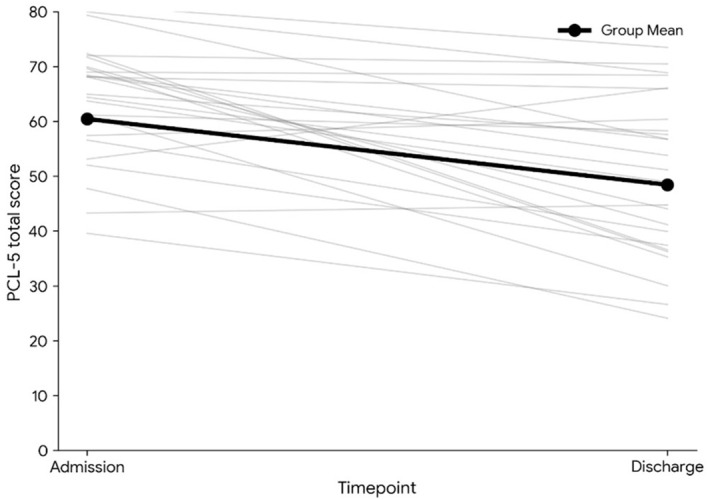
Individual and group mean changes in PTSD severity scores from admission to discharge.

Within the clinical pilot group, demographic variables were not associated with clinical change. However, length of hospitalization was positively associated with reductions in both PTSD severity, *r* = .50, *p* = .013, and dissociative symptoms, *r* = .46, *p* = .016. Paired-samples *t* tests were conducted to evaluate changes from admission to discharge (see **Figure 1**). Results indicated a significant reduction in total PTSD severity with a large effect size. Although most participants remained above the PCL-5 clinical cutoff at discharge, four participants, 16%, fell below this threshold. Examination of PTSD symptom clusters revealed significant reductions in Negative Alterations in Cognitions and Mood and Re-experiencing (see **Table 3**). Reductions in Hyperarousal and Avoidance reached nominal significance, *p* = .02 and *p* = .03, respectively, but did not survive Bonferroni correction for multiple comparisons, α = .0125. Dissociative symptoms showed a modest decrease from admission, *M* = 39.11, *SD* = 17.41, to discharge, *M* = 34.97, *SD* = 18.06; however, this change was not statistically significant, *t*(27) = 1.32, *p* = .099, with a small effect size, *d* = 0.23. Similarly, nonsignificant reductions were observed across all DES-II subscales. A mixed-design analysis of variance further indicated that reductions in PTSD symptoms occurred regardless of initial dissociative levels, *F*(1, 23) = 1.25, *p* = .275. To evaluate the fourth hypothesis, associations between change scores were examined (see **Table 4**). A significant relationship emerged between reductions in total dissociation and reductions in overall PTSD severity. Examination of dissociative dimensions indicated that decreases in Absorption were most strongly associated with overall PTSD reduction and specifically with decreases in Hyperarousal. Descriptive examination of individual trajectories revealed that 64.3% of participants (n = 18) demonstrated a decrease in Absorption scores from admission to discharge, while 35.7% (n = 10) showed an increase. In addition, reductions in total dissociation were also associated with decreases in Hyperarousal. Changes in Amnesia showed an association with reductions in Hyperarousal but were not related to other PTSD symptom clusters. In contrast, changes in Depersonalization/Derealization were not significantly associated with PTSD symptom changes. Additionally, reductions in Avoidance were not significantly related to changes in any dissociative dimension. Finally, to examine whether the association between Absorption and PTSD severity remained evident after accounting for baseline symptom levels, a linear regression analysis was conducted predicting discharge PTSD severity from discharge Absorption while controlling for baseline PTSD severity and baseline Absorption. Results indicated that discharge Absorption remained significantly associated with discharge PTSD severity after controlling for both baseline variables (*β* = 0.53, *t* = 3.43, *p* = .002), suggesting that the relationship was not attributable solely to initial levels of PTSD symptoms or Absorption.

**Table 3:** Comparison of PTSD symptoms at admission and discharge.

	*M* (*SD*) Admission	*M* (*SD*) Discharge	*t*	*P*	Cohen's *d*
PCL-5 Total	60.48 (13.02)	48.44 (14.50)	4.61	< .001*	0.92
Re-experiencing	14.81 (4.25)	11.61 (6.05)	3.53	< .001*	0.7
Avoidance	6.14 (1.73)	5.48 (2.37)	2.28	.03	0.45
NACM	22.62 (4.47)	17.51 (6.20)	4.59	< .001*	0.91
Hyperarousal	17.21 (4.68)	14.51 (6.04)	2.35	.02	0.47
DES-II Total	39.11 (17.41)	34.97 (16.81)	1.32	.197	0.25
Absorption	4.95 (1.91)	4.42 (1.89)	1.4	.172	0.26
Depersonalization\Derealization	3.6 (2.57)	3.17 (2.28)	1.09	.285	0.2
Amnesia	2.57 (1.66)	2.21 (1.84)	0.912	.37	0.176

*n* = 25 for PTSD, *n* = 28 for Dissociation. * Significant after Bonferroni correction for multiple comparisons (α = .0125).

**Table 4:** Pearson correlations between change scores (Δ) of PTSD symptoms and dissociation.

	DES-II Total Δ	Absorption Δ	Depersonalization\ Derealization Δ	Amnesia Δ
PCL-5 Total Δ	.457*	.536**	.280	.393
Re-experiencing Δ	.280	.241	.371	.157
Avoidance Δ	.017	.192	.017	-.104
NACM Δ	.342	.372	.232	.347
Hyperarousal Δ	.474*	.584**	.117	.409*

*n* = 25. Δ (Delta) indicates change scores calculated as Admission minus Discharge. *p <.05. ** *p* <.01.

## 4 Discussion

The present study employed a two-phase naturalistic design to evaluate clinical profiles and treatment outcomes among women with histories of CSA treated within ‘By Your Side’- a specialized inpatient trauma unit. This unit synthesizes specific PTSD and dissociation interventions, such as DBT for PTSD and somatic grounding, within a core relational-psychodynamic framework. This integrated approach allows diverse techniques to operate under one roof, tailored to each patient’s individual clinical needs. The first phase of the research aimed to establish the cross-sectional associations between dissociative dimensions and PTSD symptom clusters at the point of intake. The second phase served as a pilot examination of the unit’s clinical effectiveness. For this pilot, the analysis focused on a group of women who were able to complete the minimum therapeutic period of at least one month. This structural requirement was essential to ensure that the evaluation of clinical shifts followed a substantive, continuous period of intensive care, allowing the unit’s core stabilizing interventions to properly take effect.

The primary objective was to explore how dissociative dimensions relate to PTSD symptoms within a real-world clinical setting. This unit treats a population characterized by high complexity and extensive comorbidity. While many empirical studies and specialized protocols often focus on symptomatically stable populations by excluding individuals with active suicidality or self-harm (22, 46), the clinical framework of this unit is defined by an inclusive admission policy. Accordingly, this research reflects the challenges and outcomes of treatment for women presenting with severe clinical symptoms, including substantial histories of suicidal behavior, providing preliminary evidence of treatment-related changes in a highly severe trauma cohort. Consequently, this ecological framework offers valuable insights that may extend to broader, high-risk populations that remain underrepresented in empirical research.

A notable characteristic of the sample is the high level of symptomatic distress at admission. In a meta-analytic review of dissociation among victims of childhood abuse, Vonderlin et al. (2018) (10) reported a mean DES-II score of 23.5. In comparison, the current sample exhibited a more pronounced dissociative profile, with a mean admission score of 39.1. Similarly, the mean DES-II score reported in other specialized inpatient settings for complex trauma was notably lower (25, 26). While an exact comparison of PTSD severity across studies is limited by the use of different assessment tools (21), the patients’ admission mean of 60.4 on the PCL-5 reflects a substantial symptomatic load (42). In line with our initial hypothesis, baseline dissociation and PTSD severity were significantly and positively correlated. This finding supports the observation that among survivors of CSA, symptom severity manifests across both post-traumatic and dissociative dimensions, reinforcing the link between high dissociative levels and a greater overall PTSD symptom load in populations exposed to severe interpersonal trauma (12, 47). Furthermore, this robust correlation may reflect a bidirectional, self-reinforcing cycle between these constructs; Elevated post-traumatic distress may elicit dissociative responses as a defensive strategy to attenuate overwhelming affect. In turn, persistent dissociative states can disrupt emotional processing and integration, thereby maintaining or amplifying PTSD symptoms over time (48). A more granular analysis of PTSD clusters and dissociative dimensions further clarifies this clinical pattern. Although dissociation is embedded in several PTSD clusters, absorption emerged as the most salient dimension at baseline, displaying the strongest correlations, particularly with the NACM cluster. The prominence of absorption may be especially meaningful in survivors of childhood sexual abuse, as chronic early interpersonal trauma often fosters a tendency toward inward attentional narrowing and immersive internal experience. This form of imaginative involvement, characterized by deep engagement with internal stimuli such as daydreams, sensations, or memories, can function as a psychological means of disengaging from an overwhelming external reality, to the point of reduced responsiveness to the surrounding environment (45). Its strong association with NACM in our sample suggests that internalized distress, characterized by negative self-beliefs, emotional numbing, and cognitive constriction, is closely intertwined with this habitual shift toward internally focused states.

Taken together, these baseline findings address the clinical facet of our aim, namely, to characterize the in-and-out dynamics between dissociation and posttraumatic distress at presentation; the second phase extends this perspective to the therapeutic context by examining how this relationship evolves across treatment.

Regarding the second phase of the study, we evaluated symptomatic changes from admission to discharge. Preliminary results revealed an overall reduction in total PTSD severity with a large effect size. These findings may tentatively suggest that clinical improvement could be achievable within a specialized inpatient framework, even among individuals presenting with high dissociative and post-traumatic distress. Specifically, our analysis indicated that PTSD symptom reduction occurred regardless of initial dissociative severity, aligning with prior observations that high baseline dissociation does not necessarily impede treatment-related progress (21, 22).

The significant reduction in PTSD symptoms observed in this pilot supports the potential utility of an integrative therapeutic framework that combines structured stabilization with relational and somatic processing. Importantly, these clinical gains occurred alongside naturalistic adjustments to psychiatric medications. These dynamic pharmacological updates likely interacted with the psychotherapeutic interventions, together contributing to the overall symptom reduction observed at discharge. This observed improvement aligns with previous research on the efficacy of specialized inpatient programs for complex trauma, as documented in various models ranging from 6-week interventions (33, 49) to programs of 10 to 12 weeks (24, 25). However, while those studies typically operate within fixed, time-limited protocols, the current study extends this line of inquiry by examining a personalized and significantly more prolonged treatment framework, averaging 4.5 months. Our findings indicate that when the treatment timeframe is tailored to the individual needs and clinical progress of the patient (2, 50), the duration of stay may become a relevant therapeutic factor. Unlike standardized protocols that mandate uniform termination regardless of symptom trajectory, an adaptable duration may allow patients with severe, complex distress the necessary time to establish progress. Consequently, the positive association between longer stays and symptom reduction may underscore the unique clinical utility of open-ended, progress-dependent models for populations that require extended care beyond time-limited interventions. At the same time, this association should be interpreted with caution, as length of stay likely reflects a combination of treatment exposure and patient-related factors, including clinical readiness, engagement, and treatment completion. Within this sustained environment, the largest symptomatic reduction occurred in the NACM cluster. This cluster refers to a shift in the individual’s internal representation of the self and the world, often manifesting as deep-seated existential guilt, pervasive shame, and a fundamental loss of trust (51).These symptoms are frequently rooted in and exacerbated by a history of childhood abuse, where early violations of safety and attachment boundaries often lead to distorted perceptions of the self and others, with shame serving as a central, debilitating feature (52). The significance of this reduction aligns with the model proposed by Karatzias et al. (2018) (53), who emphasize that targeting negative thoughts and attachment representations holds promise in the treatment of complex trauma. Our findings may suggest that a multi-modal framework which facilitates the deconstruction of shame-based schemas and promotes the integration of dissociated self-states, effectively addresses these core cognitive and emotional disruptions. Such clinical shifts are further supported by meta-analytic evidence confirming that trauma interventions can successfully reduce a negative self-concept (54), a process identified as a key indicator of clinical progress in survivors of childhood trauma (55). Alongside the shifts in NACM, significant reduction was observed in Re-experiencing symptoms, which represent the intrusive manifestations of PTSD. A possible explanation for this decrease may be the facilitation of clinical transition from reliving the trauma, to allowing traumatic materials to be integrated into a coherent narrative, rather than being experienced as immediate and unmodulated intrusions (56). Contrary to our hypothesis and prior inpatient findings (24, 25), dissociative symptoms did not significantly decrease. This finding must be interpreted within several methodological and clinical contexts. Methodologically, the non-significant result may be attributed to the small sample size and limited statistical power, which restricted the ability to detect meaningful change in a highly symptomatic population, as well as potential ceiling effects on the DES-II that left little room for capturing measurable reductions. Clinically, it is also possible that while the unit offers a comprehensive multidisciplinary approach, its current interventions may not be optimally targeted at treating dissociative pathology per se. This complexity is further highlighted by the heterogeneous symptom trajectories within our sample; among the participants who completed the post-treatment assessment, 35.7% of women actually demonstrated a paradoxical increase in their dissociation scores at discharge. Nevertheless, the contrast between the substantial PTSD improvement and the more modest, non-significant reduction in dissociative scores remains notable. According to Steele et al. (2016) (57), dissociation in survivors of developmental trauma represents a complex, structural organization of the personality rather than a transient symptom. These entrenched patterns, characterized by a ‘phobic avoidance’ of internal states, are inherently chronic and resistant to rapid change (57). This theoretical framework is especially relevant to the current sample, where the high baseline dissociation scores indicate a more severe and entrenched defensive structure. Although the unit addresses dissociation through integrative interventions, the lack of significant change may indicate that such a foundational defense is ‘less permeable’ to change. As noted by Brand et al. (2013) (58), in cases of severe dissociative pathology, symptomatic shift typically appears in later stages of the therapeutic process. This suggests that the underlying dissociative structure may require more prolonged and targeted structural changes to achieve a measurable reduction. To examine the fourth hypothesis, associations between specific dissociative dimensions and PTSD symptom clusters were analyzed at both baseline and across treatment. At admission, all dissociative subscales; Amnesia, Depersonalization/Derealization, and Absorption, were significantly associated with total PTSD severity, reflecting a high level of global distress. In contrast, analyses of symptom change revealed a more differentiated pattern. Consistent with its prominence at baseline, absorption also emerged as the only dissociative dimension significantly associated with both overall PTSD symptom reduction and decreases in hyperarousal, suggesting a potential continuity between the clinical pattern observed at phase one and the processes underlying change. This link points specifically to the interplay between inward attentional absorption and physiological alertness, whereby reductions in internalized absorption may be accompanied by attenuation of hyperarousal. In the context of sexual trauma, the primary source of threat often shifts from the environment to the internal world. The nature of such trauma frequently involves the experience of endogenous triggers, such as somatic sensations, which are perceived as imminent threats to the self (58). In this light, hyperarousal may tentatively take on an absorptive quality, where the state of alertness appears to be redirected inward. This preliminary pattern may suggest a narrowing of attention, in which hyperarousal and absorption function as interrelated processes that parallel the individual’s reduced engagement with the external environment.

Within this clinical context, we suggest that the intensive inpatient setting may exert its therapeutic effects through mechanisms distinct from those typically emphasized in outpatient psychotherapy. Whereas outpatient one-on-one treatment often relies on deliberate inward focus, inpatient care places a greater emphasis on continuous engagement with external reality and functional demands (59, 60). The structured, high-density environment of the ward, including scheduled activities, interpersonal interactions, and daily responsibilities, requires ongoing allocation of attention to the present moment and to shared social contexts. This sustained external engagement may interfere with maladaptive cycles of inward attentional absorption by redirecting cognitive and affective resources toward functional activity and interpersonal participation (61). In parallel, the incorporation of somatic and action-oriented interventions provides opportunities for bottom-up regulation of arousal, complementing verbal and reflective therapeutic work (38). The observed link between reductions in absorption and hyperarousal at discharge are consistent with this interpretation, suggesting - albeit cautiously - that increased anchoring in present-moment functioning may be associated with diminished reliance on dissociative attentional strategies.

Beyond the specific scope of this clinical unit, these findings may carry broader implications for other therapeutic frameworks. The observed links between different dissociative dimensions and PTSD symptoms, such as the potentially stabilizing role of external engagement, highlight clinical processes that may be relevant across settings. Consequently, the practical principles observed here, including flexible treatment duration and tailored intervention matching, could potentially inform the design and optimization of other inpatient or intensive outpatient programs treating complex trauma.

### 4.1 Limitations and future directions

While the present study offers valuable insights into a clinical population that remains underrepresented in empirical research, several limitations should be noted. The modest sample size reflects the significant challenge of recruiting this specific, high-risk population. It is important to emphasize, however, that the primary intent of this study was to conduct a preliminary pilot examination to demonstrate the feasibility of the unit’s clinical model. Given the complexity of the patients, establishing that such an intensive, long-term framework can yield measurable symptomatic improvement is a necessary first step toward broader empirical validation. Nevertheless, these results must be interpreted with significant caution given the pilot nature and modest sample size of this phase. As this was a naturalistic study conducted without a control group, it is not possible to definitively attribute the observed changes solely to the specific therapeutic interventions. In the context of a specialized inpatient ward, the implementation of a control group presents substantial ethical and practical challenges, as the standard of care is uniformly provided to all admitted patients. Consequently, other factors, such as natural remission, the natural passage of time, or the removal from external stressors, may have contributed to the symptomatic shifts observed at discharge. In addition, the potential impact of psychiatric medications should be noted. Given the high psychiatric comorbidity of the sample, medication regimens were tailored individually and adjusted dynamically throughout hospitalization. While this accurately reflects real-world clinical practice, we could not statistically control for specific combinations or dosage changes, which may have interacted with the observed symptom reduction. Additionally, regarding the selection criteria for Phase 2, this sample was restricted to patients who completed the minimum requirement of the treatment program, thereby serving as a best-case feasibility and proof-of-concept evaluation under optimal treatment conditions rather than a generalized treatment-effect estimate. Nevertheless, it is worth noting that rigorous baseline comparisons between the participants who were included in Phase 2 and those who were not revealed no significant differences across baseline PTSD severity, dissociation dimensions, or any available demographic and clinical variables. This deliberate methodological decision was necessary; Testing the model’s mechanisms specifically among those who underwent the full therapeutic dosage allowed us to examine the framework’s optimal potential within this severe cohort. Furthermore, due to the modest sample size of this pilot phase, dissociation severity was examined using a categorical median-split approach to maintain statistical interpretability. Future large-scale replication studies should employ continuous mixed-effects modeling to capture more nuanced interaction trajectories. Regarding measurement, the study relied on self-report questionnaires, which may be prone to reporting biases (62); however, it is important to note that the formal diagnoses were established through comprehensive clinical psychiatric assessments upon admission. Additionally, while the International Trauma Questionnaire (63) might have more specifically captured the profile characteristic of this population, the PCL-5 was utilized as part of the unit’s routine care and ongoing clinical monitoring. Consequently, the present measurement approach focuses primarily on core PTSD symptoms and does not capture specific disturbances in self-organization (DSO), which are central to complex trauma presentations. Future research should prioritize the use of specific Complex PTSD measures, to further delineate the unique symptomatic shifts and treatment responses within this clinical profile. The speculative nature of the pilot findings, including the observed synchrony between Absorption and Hyperarousal, highlights the need for more fine-grained data in future research. Studies incorporating mid-treatment assessments would allow a more precise examination of the temporal trajectory of symptomatic change. In addition, empirical evaluation of the proposed attentional processes, for example through experimental paradigms, may help clarify whether shifts toward greater engagement with external reality contribute to clinical improvement. A mixed-methods approach integrating qualitative components could further enrich understanding of patients’ subjective experiences of change within this integrative inpatient framework. Future research should also address the absence of follow-up assessments and the limited sample size by employing larger designs with repeated measurements. Including clinician-rated measures alongside self-report instruments would strengthen methodological rigor. Such work is needed to move beyond preliminary feasibility and to better delineate processes associated with improvement and the longer-term stability of treatment effects in specialized inpatient interventions for this population.

## Data Availability

The raw data supporting the conclusions of this article will be made available by the authors, without undue reservation.
